# Pain science education and exercise interventions for people with knee or hip osteoarthritis: a systematic review, content and meta-analysis

**DOI:** 10.1186/s12891-025-09313-4

**Published:** 2025-11-22

**Authors:** Anna Louise Hurley-Wallace, Vincent Cheng, Wendy Bertram, Catherine Jameson, Vikki Wylde, Katie Whale

**Affiliations:** 1 Musculoskeletal Research Unit, Bristol Medical School, University of Bristol, Learning & Research Building, Southmead Hospital, Bristol, BS10 5NB England, UK; 2 NIHR Bristol Biomedical Research Centre, University Hospitals Bristol and Weston NHS Foundation Trust and University of Bristol, Bristol, England, UK; 3 University Hospitals Bristol and Weston NHS Foundation Trust, Bristol, England, UK

**Keywords:** Pain science education, Explain pain, Physical activity, Total knee replacement, Total hip replacement, Osteoarthritis, Content analysis

## Abstract

**Background:**

Physical activity can improve pain and wellbeing for people with osteoarthritis, including those awaiting joint replacement, for whom physical activity can improve post-operative recovery. However, people with osteoarthritis report pain as a barrier to exercise. Pain Science Education (PSE) can reframe this by addressing beliefs about pain that impact exercise. This systematic review aimed to map the content of PSE interventions for people with osteoarthritis, and evaluate the effectiveness of intervention components.

**Methods:**

This review synthesised evidence on PSE and exercise interventions for people with knee or hip osteoarthritis, including those awaiting joint replacement. Databases were searched for RCTs and non-randomised studies, from inception to 1 August 2024 (MEDLINE/PsycINFO/EMBASE/PEDro). Risk of bias was assessed (RoB 2/ROBINS-I). Intervention content was analysed using content analysis. Outcomes were analysed quantitatively, including a component network meta-analysis (cNMA) of RCTs, presented parallel to non-randomised study results. Treatment effects were evaluated against PSE intervention components to understand which components impacted outcomes (pain/physical functioning/catastrophizing/kinesiophobia/self-efficacy).

**Results:**

Twelve reports of 10 interventions (20–103 participants per study) were included. Content analysis identified six domains: introductory topics, acute pain mechanisms, chronic pain mechanisms, factors that sustain pain, multidisciplinary education, and exercise components. cNMA: PSE had little-to-no effect on pain and physical functioning, with beneficial effects on catastrophizing, kinesiophobia and self-efficacy.

**Conclusions:**

This review highlights potential effectiveness of PSE intervention components for improving psychological outcomes for people with osteoarthritis. PSE may act as a mechanism of action for exercise interventions, and is less successful delivered standalone. PSE should be delivered using relatable examples and tailored exercises to ensure accessibility. Findings should be interpreted cautiously due to the small number of heterogeneous studies.

**Systematic review registration:**

PROSPERO CRD42023488027.

**Supplementary Information:**

The online version contains supplementary material available at 10.1186/s12891-025-09313-4.

## Introduction

Total knee and hip replacements (TKR/THR) are the second and third most common elective surgical procedures in UK. Over 150,000 operations were performed in 2023 by the UK National Health Service (NHS) [1, 2], with a projected increase of up to 40% by 2060 [3]. The most frequent reason for undergoing joint replacement is osteoarthritis, indicated by chronic pain and reduced joint function [4, 5]. The benefits of low-impact physical activity for people with osteoarthritis are well-established, with improvements in pain, stiffness and quality of life indicated across a range of exercise modalities [6, 7]. Research has shown that physical activity can improve painful symptoms in up to two-thirds of people with knee osteoarthritis [8]. For people awaiting joint replacement, increased physical activity can improve overall mental and physical health pre-operatively, which can improve post-operative recovery and long-term outcomes [9, 10].

Despite the known benefits of exercise, people who experience osteoarthritis often have low levels of physical activity [9, 11]. Research has identified various barriers to exercise for people with osteoarthritis, including negative past experiences of physical activity, pain-related distress, a lack of motivation and support, and beliefs that exercise is ineffective or harmful [12]. Osteoarthritis is frequently described as ‘wear and tear’ of the joints, leading patients to believe that pain experienced during physical activity means they are further damaging their joints [13, 14], despite strong evidence to the contrary [15, 16]. Research and theory suggest that reducing this ‘fear of movement’ or ‘kinesiophobia’, is essential to improving physical functioning, and reducing pain levels [17–19].

To encourage people with osteoarthritis to be physically active, counterproductive beliefs about pain and exercise must be addressed. Such beliefs can be addressed using Pain Science Education (PSE); an intervention from the field of physiotherapy, which is typically used with patients with chronic musculoskeletal pain to encourage exercise [20]. PSE is broadly defined by its aim to help people understand that experiences of pain do not always correlate with tissue damage, and that exercising with pain can be safe and beneficial. This is done by explaining the physiological basis of pain perception to individuals and providing a scientific basis for the biopsychosocial model of pain [21–24]. PSE is frequently combined with therapeutic exercise, with the ongoing goal to establish patients’ confidence in physical activity [20].

Although PSE guidelines are available [20], there is significant heterogeneity in the content of PSE interventions that are delivered in practice, particularly when PSE is adapted beyond idiopathic chronic pain to other types of pain, such as osteoarthritis. A recent review investigated the effectiveness of neurophysiological pain education for people with knee osteoarthritis, finding reductions in pain and catastrophizing across seven studies [25]. However, educational strategies in this review were broad, including both PSE, and pain education within cognitive-behavioural frameworks. Further investigation is needed to clarify what content is being delivered within PSE interventions that target people with osteoarthritis, including those awaiting join replacement. This clarification of content can aid understanding of which components of PSE are effective for people with osteoarthritis, and separate the effects of PSE from other psychological and behavioural strategies that are commonly included in pain management interventions.

### Aims

This review aimed to map the content of existing PSE and exercise interventions for people with knee or hip osteoarthritis, evaluate the effectiveness of intervention components, and understand factors impacting intervention engagement. Specific objectives were to:

1. Identify and describe the key content of existing PSE and exercise interventions for people with knee or hip osteoarthritis.
2. Evaluate the effectiveness of different components of PSE and exercise interventions on participants’ physical and psychological outcomes.
3. Understand the level of tailoring available within existing PSE and exercise interventions (e.g., available translations, formats, and support).
4. Identify barriers and facilitators to engagement with existing interventions.

## Methods

Systematic review, content- and meta-analysis methodology were used to synthesise evidence from studies investigating PSE and exercise interventions for people with painful knee or hip osteoarthritis, who were yet to receive a knee or hip replacement surgery. This report adheres to Preferred Reporting Items for Systematic Reviews and Meta-Analyses (PRISMA) guidance [26] (Additional File 4, Additional File 5). The protocol for this review is available via PROSPERO (registration: CRD42023488027).

### Eligibility criteria

#### Population

Adults (aged over 18-years) waiting for primary knee or hip replacement, or with diagnosed painful knee or hip osteoarthritis [27] who have not yet received a replacement for the targeted joint. Studies including patients with additional multiple long-term conditions are included.

#### Intervention

##### Types

Interventions delivered to patients with painful knee or hip osteoarthritis any time prior to receiving a primary joint replacement. Interventions delivered in any format (in-person, telephone, online, hybrid).

##### Duration

Any length/duration. 

##### Content

PSE is broadly defined as educational content on pain biology and physiology, which aims to increase a patient’s understanding of the physiological processes involved in pain perception [21–24].

Eligible interventions had to be tagged using subject-specific wording: *‘pain science education’*,* ‘therapeutic neuroscience education’*,* ‘pain neuroscience education’*,* or ‘explain pain’* [22].

Standalone PSE interventions, or combined PSE and exercise interventions, that aimed to increase physical activity as part of pain management e.g., structured exercise, prescribed physiotherapy, movement or mobilization.

#### Outcomes

##### Primary

Post-intervention pain severity.

##### Secondary

Psychological factors; physical functioning scores; physical activity levels; barriers and facilitators to intervention engagement.

#### Study design

Randomised controlled trials (RCTs) and non-randomised studies, including before-and-after studies, pilot studies, feasibility studies, cohort studies, case-control studies, and time-series. Non-randomised studies were included, ensuring to account for exposures not obviously labelled as ‘interventions’, which may occur in the course of usual healthcare, for example, clinical programmes [28]. Protocols correspondent to the aforementioned study designs were included. Conference abstracts were excluded.

#### Language

Studies published in English language only. Non-English language articles were excluded per the study need to extract detailed descriptions of intervention content. The researchers extracting intervention content/design information were not fluent in other languages.

### Identification of evidence

#### Search strategy

Searches were conducted within electronic databases MEDLINE, PsycINFO, EMBASE (via Ovid), and PEDro (Physiotherapy Evidence Database), from inception to 1 August 2024. An example search strategy is provided in Additional File 1. Reference lists of relevant reviews and included studies were searched. A registry search was carried out in clinicaltrials.gov using the disease term ‘osteoarthritis’, and the intervention term ‘pain science education’, to identify ongoing trials.

#### Screening and data extraction

All identified articles were initially imported into EndNote, where duplicates were removed using the duplicate removal function. All remaining articles were then transferred into Rayyan [29], where they underwent blinded screening by two reviewers (AHW, KW), based on titles and abstracts. Conflicting decisions were discussed and resolved between the reviewers. Remaining full-text articles underwent an additional round of blinded screening by AHW and KW. Uncertain articles were confirmed for final inclusion by a third reviewer (VW).

Data on study characteristics, methodology, intervention content and participant feedback were extracted from included articles by reviewers and cross-checked by a second reviewer (AHW, VW, KW, WB). Quantitative outcome data were extracted by VC and cross-checked by AHW. All data were extracted into Microsoft Excel.

#### Risk of bias assessment

Risk of bias was assessed by two reviewers (AHW, KW) using the Cochrane Risk of Bias 2 (RoB 2) tool for randomised trials and the Risk of Bias in Non-randomized Studies of Interventions (ROBINS-I) tool for non-randomised studies [30, 31]. Traffic-light charts were generated using the *robvis* web app [32].

### Analyses

#### Content analysis and qualitative synthesis

An inductive content analysis and narrative qualitative synthesis was used to characterise the content of the PSE and exercise interventions [33]. PSE and exercise content were extracted, sorted into categories, and presented in tables (including count summaries). Due to the variety of PSE content, overarching domains were used to group discrete categories together e.g., ‘Introductory pain science topics’ are presented in one table, which contains eight discrete categories. Further detail on content analysis methodology is available in the study protocol (PROSPERO registration: registration: CRD42023488027).

All interventions were additionally summarised using Template for Intervention Description and Replication (TIDieR) checklists [34], to allow comparison of intervention delivery (as opposed to the content). Tailoring and modifications, support provided to participants, and barriers and facilitators to engagement are described in the qualitative synthesis.

#### Quantitative analysis

A component network meta-analysis (cNMA) approach was used to investigate the treatment effects of PSE within included RCTs. Pooled standardized mean differences (SMD) or mean differences (MD; if the same scale was used across studies) were summarised with 95% confidence intervals (CIs). A random-effects model was used to group similar follow-up periods when an outcome was reported by more than three studies: 1) < 8 weeks; 2) 8–12 weeks; and 3) >12 weeks. Only five outcomes between 8 and 12-weeks were synthesised: physical functioning (WOMAC total) [35–37], pain (WOMAC pain sub-scale) [35], Pain Catastrophizing Scale (PCS) [38, 39], Tampa Scale for Kinesiophobia (TSK) [40–42], and Pain Self-Efficacy using either the Chronic Pain Self-Efficacy Scale (CPSES) [43] or Pain Self-Efficacy Questionnaire (PSEQ) [44]. These outcomes were chose as the other results at other time points were sparse and unsuitable for meta-analyses.

For the main quantitative analysis, interventions were categorised based on the PSE domains identified in the content analysis, excluding the exercise components. Interventions were coded according to whether they were ‘high’, ‘low’ or contained ‘some’ aspects within each PSE content domain (D1 to D5) (see Fig. 2). This approach was selected as PSE presented varying components and may violate the transitivity assumption of network meta-analysis. However, the limited number of studies for each outcome and diversity of interventions resulted in difficulties assessing heterogeneity. Hence, an additive network meta-analysis model [45] was employed to connect common components, such as ‘usual care’, which was used to describe conventional education programs used as comparators across studies. This allowed illustration of the potential effects produced by different components of the PSE interventions.

Forest plots of cNMA were presented parallel to individual study results of non-RCTs [46] and observational studies [47, 48]. Whilst meta-analyses of non-RCTs and observational studies were not conducted due to small numbers of studies, their results were presented alongside the cNMA of RCTs to identify PSE key components. Where mean and standard deviation (SD) were not reported, values were estimated following recommendations by the Cochrane Handbook [49] and Shi et al. [50]. Supplementary data was used to compute the WOMAC score for one study [48].

Two sensitivity analyses were conducted. In the first, effect sizes were estimated using post-intervention values to assess the statistical assumptions. In the second, we combined different types of PSE interventions in the network meta-analyses to improve the statistical power (see Additional File 5 for further details). All analyses were conducted using R version 4.3.2 on RStudio 2023.06.2 + 561. The meta-analysis model was based on a frequentist inference approach. Pooled estimates and forest plots were generated using *netmeta* and *metafor* packages.

## Results

### Included studies

**Fig. 1:**
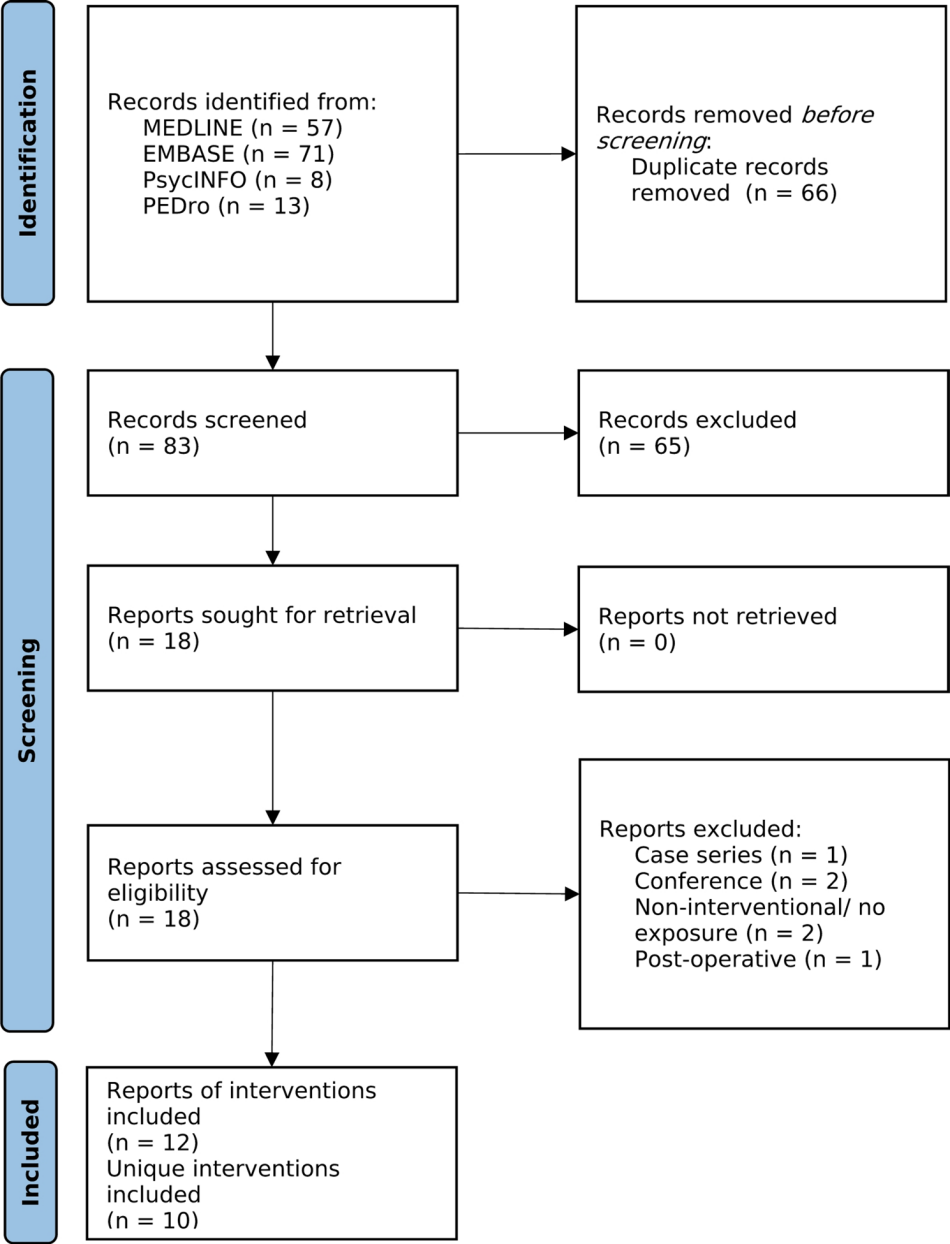
PRISMA flow diagram for articles identified via electronic databases

Searches identified 149 articles which after removal of duplicates yielded 83 potentially eligible articles. After screening, 12 articles were included, relating to 10 unique interventions (Fig. 1).

#### Other sources

The registry search for ongoing trials identified one study conducted with knee osteoarthritis patients, in a Turkish hospital, that was completed on 7 December 2023 (NCT05649995). No associated publications were available. Reference lists did not reveal any additional articles for inclusion.

**Table 1:** Study characteristics for included interventions (*n* = 10)

Author (year), Study Registry	Study Period	Country	Study Design	*N* recruited	Age (Mean ± SD)	%F	Setting	Pain at baseline (Mean ± SD)	Condition	Intervention	Follow-up
Lluch (2018) [56]; Huysmans (2021) [secondary analysis] NCT02246088	Jan 2014 to Feb 2015	Spain	RCT	54	70.25 ± 6.7	63.6	Orthopaedic presurgical outpatients	5.8 ± 1.55 NPRS [0–10]	TKR presurgical	PSE + Physiotherapy: knee mobilizations (*n* = 22) Usual care: Biomedical education (*n* = 22)	8 weeks, 12 weeks, 3 months after surgery
Rabiei (2023) [53] IRCT20210701051754N1	Jul 2021 to Mar 2022	Iran	RCT, Pilot study	54	60.5 ± 5.6	40.7	Physiotherapy outpatients	10.65 ± 3 WOMAC (pain) [0–20]	Knee osteoarthritis	PSE + Exercise: Pilates (*n* = 27) Exercise only: Pilates (*n* = 27)	8 weeks
Stanton (2020) [24] ACTRN12618001149257	Jul 2018 to Feb 2019	Australia	RCT, Feasibility study	20	67 ± 7.4	70.0	Community/mixed outpatients	11.5 ± 3.3 WOMAC (pain) [0–20]	Knee osteoarthritis	PSE + Exercise: graded walking (*n* = 10) Exercise: graded walking + Standard education about knee osteoarthritis/physical activity and sham ultrasound sessions (*n* = 10)	4 weeks, 8 weeks, 26 weeks
Supe (2023) [54]	Aug 2021 to Jun 2022	India	RCT	70	58.43 ± 5.73	91.0	Physiotherapy outpatients	6.31 ± 1.375 NPRS [0–10]	Knee osteoarthritis	PSE + Physiotherapy (conventional) (*n* = 35) Physiotherapy only (conventional) (*n* = 35)	2 weeks
Terradas-Monllor (2023) [17] NCT03198247	Sep 2019 to Jun 2020	Spain	RCT, Feasibility study	33	72.25 ± 5.1	72.2	Early presurgical inpatients		TKR presurgical	PSE + Physiotherapy (Coping Skills Training) (*n* = 11): PSE + Physiotherapy (multimodal including, supervised exercise, Coping Skills Training and osteopathic therapy) (*n* = 8): Usual care: Biomedical education + pre and post-operative physiotherapy (*n* = 14)	8 weeks
Louw (2019) [46] NCT03231566	Jul 2015 to Feb 2017	USA	Non-randomised controlled trial	103	71.7 ± 10.3	58.3	Orthopaedic presurgical outpatients	4.7 ± 2.2 NPRS [0–10]	TKR presurgical	PSE (*n* = 49) Usual care (*n* = 54): Standard preoperative education	1 month, 3 months, 6 months
Modarressi (2023); Modarressi (2023) [Protocol] NCT04954586	Not reported	Canada	Before-and-after	19	63.3 ± 10.5	73.0	Community/mixed outpatients	Median (range) 41.7 (11.1–83.3) KOOS (pain)	Knee osteoarthritis	PSE + Exercise: group strength and balance exercises (Yoga-based) (*n* = 14)	8 weeks
Preece (2021) [48]	Not reported	UK	Before-and-after, Pilot study	21	60 ± 9	45.5	Physiotherapy outpatients	8.2 ± 2.4 WOMAC (pain) [0–20]	Knee osteoarthritis	PSE + Physiotherapy: functional muscle retraining (*n* = 11)	12 weeks, 9 to 15 months
Gholami (2023) [52] [Protocol only]	N/A	Iran	RCT	129 Expected	N/A	N/A	Rheumatology outpatients	N/A	Knee osteoarthritis	PSE only (*n* = 32) Physiotherapy: blended exercises (*n* = 32) PSE + Physiotherapy: blended exercises (*n* = 32) Control group: Education about the knee joint (*n* = 32)	3 months, 6 months
Stanton (2021) [55] [Protocol only]	N/A	Australia	RCT	198 Expected	N/A	N/A	Community/mixed outpatients	N/A	Knee osteoarthritis	PSE + Exercise: graded walking and strengthening (*n* = 99) Exercise: graded walking and strengthening + Standard education about knee osteoarthritis (*n* = 99)	1 week, 12 weeks, 6 months, 12 months

### Study characteristics

Studies included full and pilot/feasibility RCTs (*n* = 5) and non-randomised designs (*n* = 3). Two interventions were described in protocols only. Studies were conducted in Spain, Iran, Australia, India, the USA, and Canada (see Table 1). Sample sizes varied from 20 to 103 participants. Participant mean age ranged from 58 to 72 years. The percentage of female participants ranged from 41% to 91%.

### Summary of intervention characteristics

Interventions contained a variety of PSE content, ranging from detailed neuroscience education to coping and behavioural skills. PSE was used to facilitate physical exercise and/or physiotherapy (*n* = 9), with the exception of one presurgical PSE study [51]. PSE and exercise interventions were delivered by trained practitioners, with exercise components delivered by physical therapists (*n* = 7), yoga therapists (*n* = 1) [47], and sports therapists (*n* = 1) [52]. Most interventions were delivered in clinical settings(*n* = 8). Two interventions were conducted entirely in-person at outpatient clinics [51, 53], whereas six interventions were delivered partially in clinical settings with additional remotely-delivered components(*n* = 6). Remotely-delivered components included group telerehabilitation [54], educational booklets [24] and instructional videos [47]. One intervention was delivered solely at participants’ homes [17], and one was delivered by remote telerehabilitation only [52]. In-person sessions were held both individually(*n* = 7) [17, 24, 48, 51, 54–56] and in groups(*n* = 2) [47, 53]. Six interventions were delivered in English language [24, 47, 48, 51, 54, 55], two in Persian [52, 53], and two in Spanish [17, 56].

### Content analysis and qualitative synthesis

Ten intervention descriptions, including two protocol-only descriptions [24, 52], were included in the content analysis of PSE and exercise interventions. Six overarching content domains (D) were identified:

- D1: Introductory pain science topics.
- D2: Acute pain mechanisms.
- D3: Chronic pain – neurophysiological mechanisms.
- D4: Factors that sustain chronic pain.
- D5: Multidisciplinary (MDT) pain management education.
- D6: Exercise components.

PSE domains D1 to D4 were generated in alignment with practice guidelines for using PSE to explain pain to patients with chronic pain [20]. Two interventions referenced these guidelines as shaping their PSE content [53, 56]. The PSE and pilates intervention [53] stated that PSE content was based solely on this guidance. D5 refers to multidisciplinary education, where interventions included additional education or advice delivered adjunctively to the core PSE topics (D1 to D4).

#### D1: introductory pain science topics

Introductory pain topics were presented in nine out of 10 interventions, grouped into 8 content categories. The most covered topic was the explanation that pain is a perception or interpretation (rather than a sensation) (*n* = 7), followed by the explanation of pain as a protective function or ‘Protectometer’ (*n* = 6) [55]. Five interventions gave a definition of pain or covered ‘What is pain?, and four described the distinction between acute and chronic pain. Basic nervous system anatomy was covered by two interventions [24, 55]; with both interventions using a participant handbook to expand on PSE topics. Two interventions presented case examples to aid understanding of pain as a perception (e.g., phantom limb pain) [17, 24]. One intervention did not include introductory topics and began with acute pain mechanisms [51]. A breakdown of introductory PSE topics is provided in Table 2.

**Table 2:** PSE content summary for categories relating to ‘Introductory pain science topics’. *n*=8 categories. Content categories are coloured using a traffic light system, where green = topic covered by intervention, red = topic not covered by intervention. Count summaries indicate how many interventions covered each topic

#### D2: acute pain mechanisms

Mechanisms of acute pain were covered in across all interventions, grouped into 12 content categories (Table 3). The level of neuroscience detail varied across studies, for example only one study mentioned ion gates. Interventions that covered the most (70% to 90%) of the acute pain physiology topics were two pre-operative interventions [51, 56] and the PSE and pilates programme for knee osteoarthritis [53]. Descending and/or ascending neural pathways (also termed ‘upregulation’ and ‘downregulation’) was the most covered content category (*n* = 8), followed by explanations of nociception versus pain perception (*n* = 7), and explanations of the role of the brain or specific brain areas when experiencing pain (*n* = 7). One unique lay explanation used was the ‘drug cabinet in the brain’, referring to endogenous versus exogenous medication [24].

**Table 3:** PSE content summary for categories relating to ‘Acute pain mechanisms’. *n*=12 categories. Content categories are coloured using a traffic light system, where green = topic covered by intervention, red = topic not covered by intervention. Count summaries indicate how many interventions covered each topic

#### D3: chronic pain – neurophysiological mechanisms

Neurophysiological mechanisms were covered in eight interventions, grouped into seven content categories (Table 4). The most covered content related to explanations of central sensitization (*n* = 8), and references to hyperprotective states (*n* = 7), including the ‘Protectometer’ [55], hyperalgesia [52], ‘nerve sensitivity’ [51] or ‘states of alarm’ [17]. One pre-operative intervention related heightened nerve sensitivity to past surgical experiences [56]. Five interventions contained content on plasticity of the nervous system, or bioplasticity [24, 51, 53, 55, 56]. Pain modulation and modification was covered by two interventions [53, 54], one of which was the PSE and pilates intervention, which did not directly report content descriptions [53], instead referring to guidelines by Nijs et al. [20]. Specific reference to the ‘pain neuromatrix’ was made in three interventions [17, 53, 56].

**Table 4:** PSE content summary for categories relating to ‘Chronic pain – neurophysiological mechanisms’. *n*=7 categories. Content categories are coloured using a traffic light system, where green = topic covered by intervention, red = topic not covered by intervention. Count summaries indicate how many interventions covered each topic

#### D4: factors that sustain chronic pain

Factors which sustain chronic pain were included in eight interventions, grouped into five content categories focusing on the role of emotions, cognitions, pain-related behaviours and body-wide inflammation in the maintenance of chronic pain (Table 5). Four interventions included content on all identified psychological and behavioural factors: emotions/stress, cognitions, and pain behaviours [24, 48, 53, 56]. Individually, these content categories (emotions, cognitions and behaviours) were each covered by six interventions. Two interventions specifically stated their goal to integrate behavioural or mind-body aspects within PSE [48, 53]. A key concept referred to was ‘fear of movement’ or ‘kinesiophobia’. This relates to the Fear-Avoidance model of chronic pain maintenance [18], which stipulates that painful movements are often avoided, resulting in musculoskeletal deconditioning, and prolonged pain. The role of body-wide inflammation in chronic pain maintenance was emphasised in one protocol [55]. Correcting compensatory movement patterns (postural deconstruction) was a focus of the Integrated Behavioural Intervention [48].

**Table 5.** PSE content summary for categories relating to ‘Factors that sustain chronic pain’. *n*=5 categories. Content categories are coloured using a traffic light system, where green = topic covered by intervention, red = topic not covered by intervention. Count summaries indicate how many interventions covered each topic

#### D5: multidisciplinary pain management education

Additional multidisciplinary education and advice was integrated alongside the core PSE education topics (D1 to D4) in nine interventions, grouped into eight categories (Table 6). These topics were largely intervention-specific. The most covered category was discussing and challenging views of pain and exercise (*n* = 5), which goes beyond identifying pain-related cognitions to include context and discussion of experiences between practitioners and peers [24, 48, 52, 54, 55]. Three interventions gave an outline of evidence-based multidisciplinary strategies available for pain management [17, 24, 52], for example, Stanton et al. [24] explained ‘the place of pills’ in PSE session 10. A focus on transferring knowledge to adaptive behaviour change was mentioned by three interventions [48, 55, 56]. Behaviour change strategies appeared integrated throughout these interventions, with one intervention, Lluch et al. [56], specifically including adaptive behaviour change as a contents header. The EPIPHA-KNEE protocol [55] sought to elicit participant reflections on their confidence in moving.

**Table 6:** PSE content summary for categories relating to ‘Multidisciplinary pain management education’. *n*=8 categories. Content categories are coloured using a traffic light system, where green = topic covered by intervention, red = topic not covered by intervention. Count summaries indicate how many interventions covered each topic

Two interventions challenged views around the use of X-rays as a diagnostic tool for osteoarthritis. This was a specific strategy used in the Stanton et al. interventions [24, 55], reinforcing the core message of PSE; that pain is not a marker of tissue damage. Reconceptualising views of post-operative pain was covered by two of the preoperative interventions [51, 56].

Three interventions included an explanation of exercise progression and how this can change pain levels over time, with lay references to ‘the sweet zone’ [55] and ‘nudging the edge’ of pain [47]. The pre-operative multimodal physiotherapy intervention [17] specifically stated coverage of: ‘The exercise influence on pain – neurophysiological effects of therapeutic exercise’. The Blended Exercises intervention [52] included additional ‘healthy lifestyle’ guidance. This consisted of nutritional advice, self-management education, and encouragement to be more active. One intervention used biofeedback techniques to show participants’ muscle activity [48].

#### D6: exercise components

Nine interventions contained exercise or physical activity components Interventions covered a breadth of exercises which were conceptualised as general ‘Physical exercise’ and ‘Physiotherapy’ programmes. The only intervention that did not include exercise was the pre-operative PSE session by Louw et al. [51]. To evaluate intervention components, per the aims of this review, activities were split into two categories: (i) ‘Physical exercise’, which included pilates, yoga-based classes (strength and balance), and graded walking, and (ii) ‘Physiotherapy’, which included targeted physiotherapy exercises, joint mobilizations, and functional muscle retraining. A further breakdown of exercises in each intervention can be viewed in Additional File 2.

Exercise components were predominately designed to be home-based. Several interventions focused on knee joint exercises, including knee flexion/extension (*n* = 4) [17, 47, 54, 56] and step-ups (*n* = 4) [17, 24, 47, 52]. A selection of functional, home-based exercises such as progressive walking, wall squats, and pelvic lifts/bridges, were included in eight interventions. The only PSE and exercise intervention that did not utilise home-based exercises was the pilates programme [53], which used in-person, group exercise sessions. The Pain Informed Movement programme conducted group ‘neuromuscular’ exercise sessions (strength and balance), carried out twice weekly in-person with a Yoga instructor, and once weekly at home [47, 57]. Four interventions described breathing exercises as part of their exercise component [17, 47, 48, 52].

### Tailoring and additional support

Tailoring elements were described in four interventions, which all personalised their exercise programmes by participants’ ability or progress. This included a tailored walking programme [24], a protocol for a tailored walking plus strengthening programme [55], personalised muscular exercises [48], and a protocol for a tailored blended exercise programme [52]. In addition to tailoring for exercise ability, one protocol described tailoring educational content to participant circumstances, using the participant’s own wording to explain concepts as much as possible [55].

No tailoring or modifications were made to provide translations, alternative formats, or additional support for people from diverse cultures or backgrounds, or neurodiverse individuals.

### Intervention engagement

Engagement in terms of adherence was reported in three interventions [24, 47, 53]. Results indicated that participant engagement was high (78% to 96%) for various intervention components, including workbook activities [24], home exercises [47], and in-person exercise sessions [24, 53].

Three feasibility studies [24, 47, 48] sought qualitative feedback from intervention participants. This was done via co-design workshops [48], short surveys and telephone calls [24] and focus groups [47]. Key barriers and facilitators to participant engagement are summarised below.

#### Barriers

- Difficulty understanding PSE content and concepts; information was too ‘full-on’ [24, 47].
- Lack of practical examples of how to apply of knowledge and techniques [24, 47].
- Too much information; not all topics apply to all people [24].
- Lack of programme flexibility (no weekend appointments) [24].
- Undertaking other treatments [24].
- Not wanting to have an x-ray of the joint [24].

#### Facilitators

- Friendly, encouraging and positive practitioners [24, 47, 48].
- Inclusion of videos and animations [47, 48].
- Focus on goal setting [24].
- Programme flexibility and home-based tasks [24].
- Well-spaced information (small bites info) [24].
- Personalised feedback and tailoring [47].
- Framing of the programme as holistic and patient-centred [48].
- Exercising as a group as opposed to individually [24].

### Quantitative analysis of RCTs and non-randomised studies

**Fig. 2:**
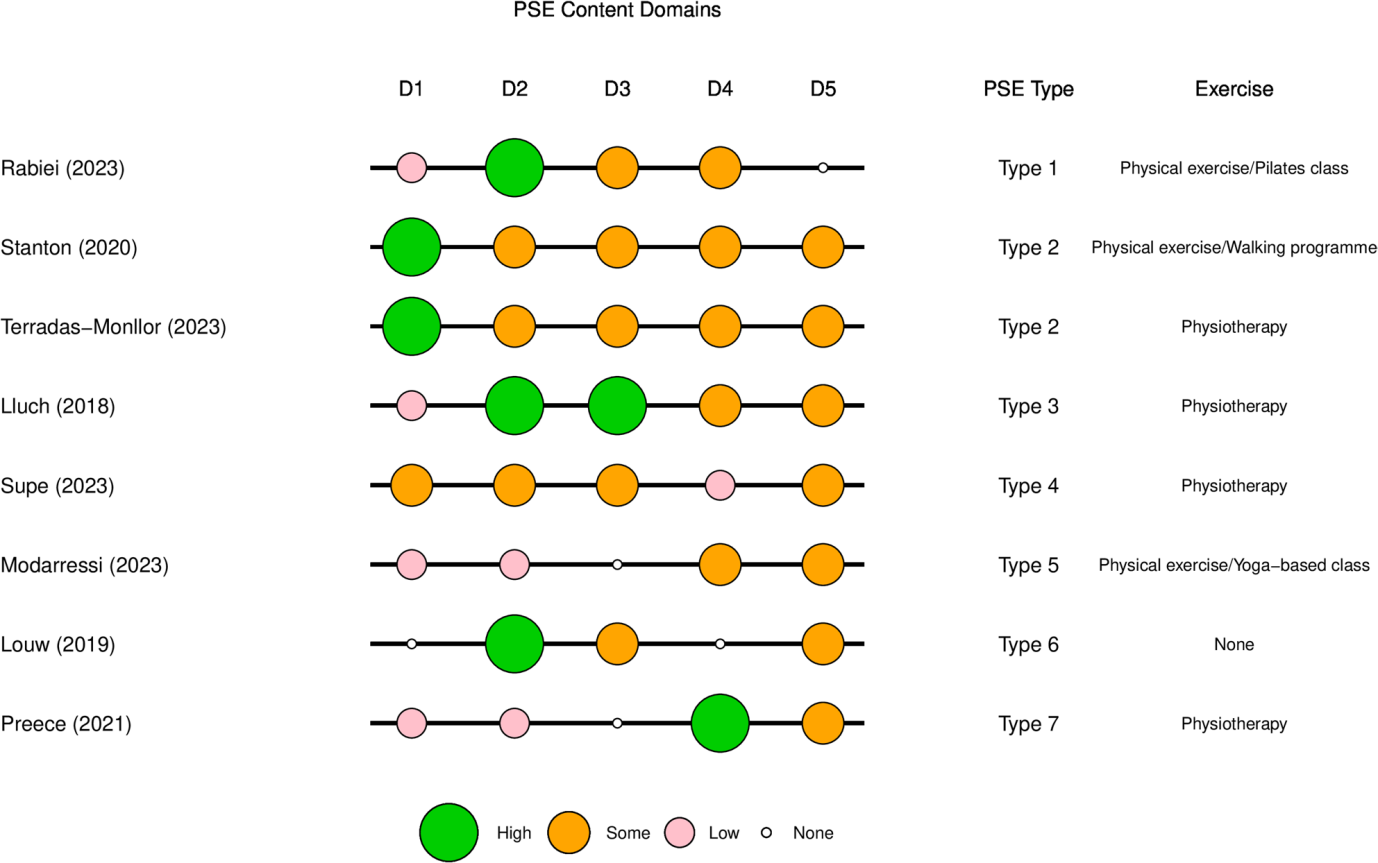
Bubble plot summary of PSE and exercise interventions delivered in RCTs and non-randomised studies (*n*=8)

Eight studies were included in the quantitative analysis for identifying key PSE intervention components (RCTs *n* = 5, non-randomised studies *n* = 3). The PSE and exercise intervention content delivered in these studies is summarised into components or ‘domains’ for analytical purposes (Fig. 2). Interventions are scored ‘low’, ‘some’, or ‘high’ in each PSE domain from the content analysis (D1 to D5), and a brief description of the exercise component for each study is given. Two RCTs [17, 24] were grouped together as ‘PSE Type 2’, as overarchingly these interventions had similar PSE content. This grouping allowed results to distinguish between the effects of PSE depending on the type of exercise that was delivered adjunctively (i.e., graded walking versus multimodal physiotherapy).

**Fig. 3:**
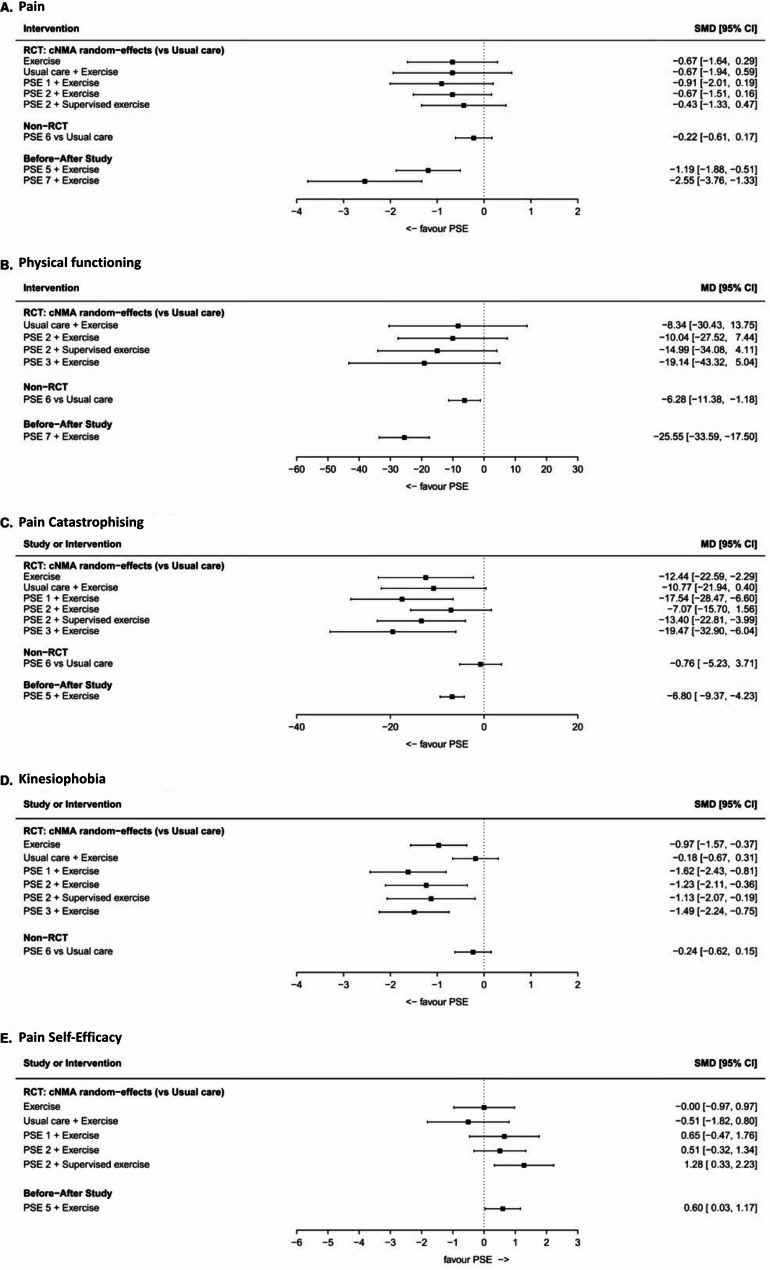
Combined forest plots of outcomes for RCTs and non-randomised studies (*n*=8)

The quantitative results presented in in Fig. 3 show the relative treatment effects of different combinations of PSE and exercise on five outcomes (A to E) for the 8 to 12 week timeframe.

Implications for the effectiveness of PSE for the primary and secondary outcomes for the 8 to 12 week timeframe (see Fig. 3) are summarised below.

#### Treatment effects by intervention content

Pain (measured by the WOMAC pain sub-scale) – PSE had little-to-no effect on pain outcomes based on the RCT evidence. SMDs ranged from − 0.91 (95% CI: −2.01 to 0.19) to −0.43 (−1.33 to 0.47), with all estimates favouring the interventions of PSE and exercise but with confidence intervals crossing zero (Fig. 5A). However, exercise of any type may have direct, beneficial effect on pain, although the evidence is not conclusive (SMD − 0.67, 95% CI: −1.64 to 0.29). Before-and-after study evidence suggests moderate improvements in pain (SMDs: −1.19 [95% CI: −1.88 to −0.51] by Modarresi et al. [47] and − 2.55 [95% CI: −3.76 to −1.33] by Preece et al. [48]), although this should be interpreted cautiously due to the study designs.

Physical functioning (measured by the WOMAC total score) – RCT evidence suggests PSE had little-to-no effect on overall physical functioning (SMDs ranged from − 19.14 (95% CI: −43.32 to 5.04) to −10.04 (95% CI: −27.52 to 7.44), Fig. 3B). However, interventions that contained a greater amount of PSE content from D2 and D3 (acute/chronic pain mechanisms) elicited more positive change in physical functioning compared to those that included less D2 and D3 content. Observational study evidence suggests moderate improvements in physical function (SMDs: −6.28 (95% CI: −11.38 to −1.18) for non-RCT and − 25.55 (95% CI: −33.59 to −17.50) for before and after study, although should be interpreted cautiously due to the study designs used.

Pain Catastrophising (measured by the Pain Catastrophising Scale) – PSE may have a beneficial effect on pain catastrophizing (Fig. 3C). Results imply that D1 categories (introductory pain science) combined with multimodal physiotherapy (supervised exercise and coping skills), as in Terradas-Monollor et al. [17], might be important to improving catastrophizing outcomes (MD −13.40 (95% CI: −22.81 to −3.99)) in comparison to Stanton et al. (MD −7.07 (95% CI: −15.70 to 1.56)).

Kinesiophobia (measured by the Tampa Scale for Kinesiophobia [TSK]) – PSE and exercise have some beneficial effect on kinesiophobia (Fig. 3D). D1 and D4 content (introductory pain science/factors that sustain pain) exist prevalently in RCTs that reported improvements in kinesiophobia (SMDs ranged from − 1.62 (95% CI: −2.43 to −0.81) to −1.13 (95% CI: −2.07 to −0.19)). However, the non-RCT study by Louw et al. [51] has less conclusive results (SMD − 0.24 (95% CI: −0.62 to 0.15)), implying D1 and D4 content might be less important in improving kinesiophobia when PSE content is delivered standalone, without the addition of exercise.

Pain Self-Efficacy (measured by either the Chronic Pain Self-Efficacy Scale (CPSES) or Pain Self-Efficacy Questionnaire (PSEQ)) – PSE may have a small beneficial effect on pain self-efficacy outcomes (Fig. 3E); the effect might be enhanced synergistically by exercise, especially multimodal physiotherapy (supervised exercise and coping skills, SMD 1.28 (95% CI: 0.33 to 2.23)) [17].

#### Outcomes not included in the quantitative analysis

There were a variety of outcomes reported at < 8-weeks, including pain, physical functioning and psychological outcomes. The study by Supe et al. [54] only included post-test (2-week) results. This study used different outcome measures to studies included in the meta-analysis; pain outcomes included a Numerical Rating Scale (NRS), as well as the PCS. Physical functioning (impact of condition on functioning) was measured using a Patient Specific Function Scale. All three outcome measures were found to significantly improve in the PSE group compared to conventional physiotherapy alone (pain NRS; M difference 2.51, 95% CI, 2.04 to 2.98; *p* <.0001, PSFS; M difference − 1.59; 95% CI, −1.95 to −1.22; *p* <.0001, PCS M −0.714; 95% CI, −2.28 to 0.85; *p* =.36). Stanton et al. [24] also assessed clinical outcomes at 4-weeks, including the WOMAC (overall score for physical functioning, and pain sub-scale). They found significant within-group change (PSE group) from baseline for the pain sub-scale, −3.9 (95% CI: 6.7 to −1.1), but not for the WOMAC total − 15.3 (95% CI: −24.4 to −6.2). The TSK (brief version adapted for osteoarthritis) showed zero change in fear of movement at 4-weeks, and the PCS showed no significant change at 4-weeks (2.9, 95% CI: −3.9 to 9.8).

Two studies included long-term outcomes >12-weeks that were not included in the meta-analysis. First, Stanton et al. [24] re-measured all clinical and functional outcomes at 26-weeks, including the WOMAC, TSK, PCS, and PSEQ. The only significant within-group change (PSE group) was on the PSEQ (pain self-efficacy); 8.7 (95% CI: 0.1 to 17.4). Exploratory comparisons indicated that PSE reduced unhelpful pain beliefs, and had positive effects on pain and functioning function, which were strongest at the 8-week timepoint. Lluch et al. [56] also included long-term follow-up measures 3-months after knee replacement surgery (whereas the 8-week timepoint included in the meta-analysis was pre-operative). For both the PSE + knee mobilisation group and the control group (knee mobilisation only), the WOMAC score decreased 3-months after surgery compared with baseline (PSE: −58.3%±21.9%; control: −38.6%±31.5%, *p* <.001). In the PSE group, there was a significant reduction in PCS scores 3-months after surgery, compared with the baseline scores. PCS scores at 3-months were also significantly lower in the PSE group compared to the control group (PSE: 6 ± 5.3; control: 22.7 ± 13, *p* <.001). TSK score in the PSE group significantly decreased 3-months after surgery compared to baseline, and was significantly lower compared with the control group (PSE: 21.5 ± 5.1; control: 30.8 ± 6, *p* <.0001).

#### Sensitivity analyses

Sensitivity analyses using different statistical assumptions showed similar quantitative results, although treatment effects were more conclusive for physical functioning, pain catastrophizing, and pain self-efficacy. However, these results did not account for differences in baseline values. The second sensitivity analysis, which combined different types of PSE, suggested that PSE had general additive effects on exercise, compared to exercise alone. However, most of these results were not conclusive due to small sample sizes, except for kinesiophobia and pain self-efficacy. Interventions delivered to patients with painful knee or hip osteoarthritis anytime prior to receiving a primary joint replacement. Further details of these results are provided in Additional File 5.

#### Risk of bias results

**Fig. 4:**
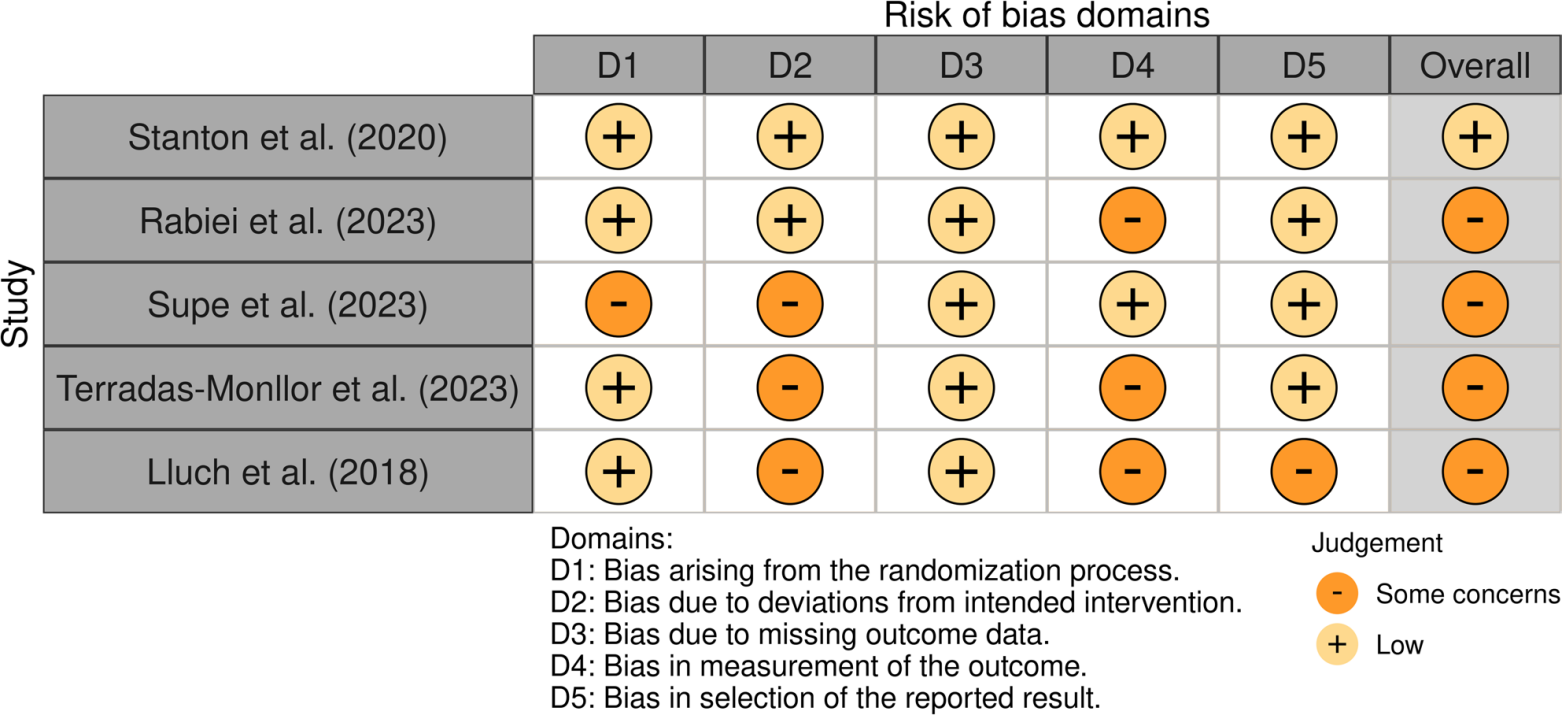
RoB 2 traffic-light plot for RCTs (*n*=5) included in review of Pain Science Education (PSE) interventions for people with osteoarthritis

Risk of bias was scored ‘low’ overall for one RCT [24], and ‘some concerns’ overall for four RCTs [17, 53, 54, 56] (Fig. 4). RCTs were generally at low risk for most domains, especially missing outcome data (D3) and reporting bias (D5). However, deviations from intended interventions (D2) and measurement of outcomes (D4) raised concerns in 3 of 5 studies, which contributed to overall scores.

**Fig. 5:**
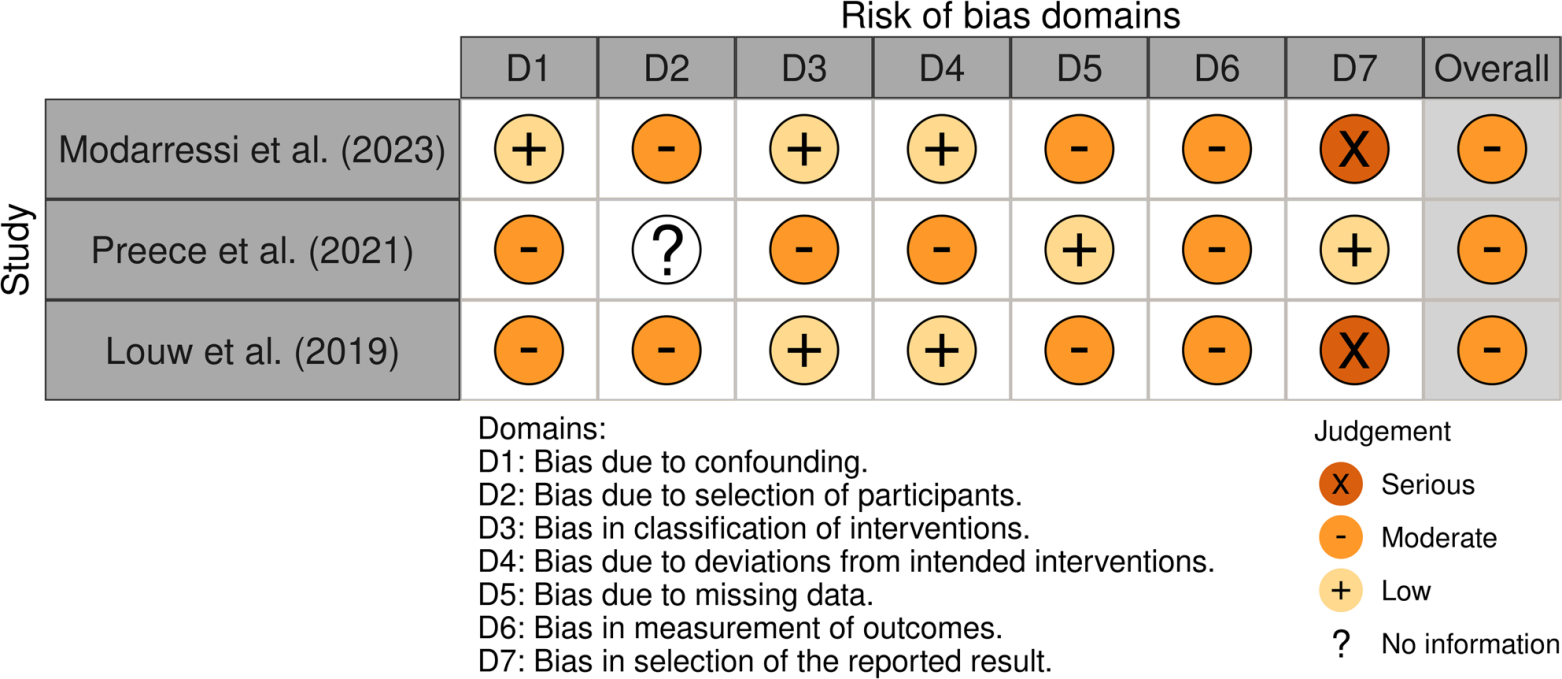
ROBINS-I traffic-light plot for non-randomised studies (*n*=3) included in review of Pain Science Education (PSE) interventions for people with osteoarthritis

Risk of bias was scored ‘moderate’ overall for all non-randomised studies [48, 51, 57] (Fig. 5). Across domains, non-randomised studies generally had moderate risk of bias, particularly in measurement of outcomes (D6) and selection of reported results (D7). Two studies had serious concerns in D7 [51, 57], indicating selective reporting. Confounding (D1), participant selection bias (D2) and missing data (D5) were also issues in 2 of 3 studies.

## Discussion

This systematic review synthesised the content and evaluated the effectiveness of diverse components of PSE and exercise interventions for people with knee or hip osteoarthritis. Meta-analysis results indicated that PSE had little-to-no effect on pain and physical functioning, with some beneficial effects on pain-related psychological outcomes; catastrophizing, kinesiophobia and self-efficacy. Results indicate that whilst PSE can reduce fear and anxieties related to pain and exercising, these psychological improvements do not translate into more physical activity or less pain. The most effective components of PSE remain unclear; due to the diversity of PSE and exercise content and statistical power of quantitative results, more trials are needed. The content analysis identified a clear need to simplify and tailor PSE content and exercises.

Despite low statistical power among the quantitative results, key intervention components contributed to small but significant positive treatment effects for psychological outcomes. In particular, combining ‘introductory pain science topics’ with ‘factors that sustain pain’ may support improvements in kinesiophobia, but only when these PSE components are delivered with an exercise component. Therefore, PSE could be conceptualised as a mechanism of action, where the intervention is the physical exercise component.

Whilst PSE had little-to-no effect on physical outcomes in the 8-to-12-week timeframe, improvements in psychological outcomes signal potential for effectiveness in overcoming barriers to exercise for people with knee osteoarthritis. Theory suggests that improvements in pain catastrophizing and kinesiophobia can interrupt the Fear-Avoidance cycle, thereby improving pain and physical functioning in the longer-term [17–19]. It is unclear how much movement on psychological outcome scales would be needed increase motivation to exercise, and the duration of post-intervention follow-up required for psychological improvements to translate to physical improvements. More RCTs to detect differences in longer-term outcomes (>3 months) are needed, particularly for pre-operative interventions [58]. A recent RCT of pain neuroscience education for patients undergoing TKR, delivered in 2-sessions pre- and post-operatively, similarly found no improvements in physical or psychological outcomes at 3-month follow-up, with the exception of ‘attention to pain’ [59].

Conversely to the findings in this review, a recent review of neurophysiological pain education for people with knee osteoarthritis found improvements in pain and catastrophizing [25]. The review included two PSE interventions evaluated herein [51, 56], plus five other interventions, which included pain education within cognitive-behavioural approaches. Cognitive-behavioural therapy is an evidence-based strategy, which is included in the NICE guidance for managing chronic pain [60]. The inclusion of cognitive-behavioural approaches in the alternative review may explain the finding of improved pain. Other research on the effectiveness of cognitive-behavioural therapy combined with exercise for musculoskeletal pain found mixed results for pain outcomes [61].

The current review summarised tailoring and support provided within PSE and exercise interventions, to aid understanding of the level of accessibility and personalisation of existing interventions. Four PSE programmes that tailored exercises based on participant progress were identified. In particular, the graded walking programme by Stanton et al. [24] was received positively by participants, who praised its flexibility and the ease of the exercises being home-based. This reflects recommendations for managing osteoarthritis, which highlight there is no clear priority for one type of exercise over another and that the choice, duration, and intensity of physical activity should be personalised to maximise accessibility and adherence [7]. Flexibility in the choice of exercise and how exercises are delivered are paramount to ensure accessibility, including to those who are underserved in healthcare, and should be incorporated into future interventions.

A major critique of the PSE interventions evaluated in this review was that the content was too ‘full-on’, being difficult for participants conceptually, and containing too much content overall. PSE is by nature a scientific explanation, however, key concepts can be communicated in plain language, such as using the ‘sweet zone’ to describe exercise tolerance or the ‘Protectometer’ to describe how pain is part of the body’s alert system. Using video explanations and case examples of people with chronic pain can make scientific concepts more accessible. It is important that future PSE interventions identify key messages and translate these into a format that patients can relate to. To do this, robust and inclusive intervention development processes are needed, where interventions are developed with patients as equal partners in research using co-design methods. Co-designing interventions with end-users is known to enhance intervention acceptability [62, 63]. This review found very limited evidence of co-design methods used to develop PSE interventions; only one behavioural intervention [48] included consultations with patients and physiotherapists throughout development.

This review sought to include studies of people with knee or hip osteoarthritis, yet only retrieved a small number of studies involving people with knee osteoarthritis, and none involving people with hip osteoarthritis. This highlights a paucity of studies investigating the effectiveness of PSE interventions for people with osteoarthritis, including those undergoing elective joint replacement surgery. Priority setting has identified the need to understand the most effective pre-operative patient education, support and advice for managing symptoms whilst waiting for TKR/THR, and for improving outcomes following surgery [64, 65]. PSE offers an avenue for further research as a pre-operative educational approach that can be used to engage people in physical activity [13, 14]. Physical activity is an important part of pain and symptom management for people waiting for joint replacement, as it reduces their risk of functional decline [9, 10] and poor post-operative outcomes, such as chronic post-surgical pain [66]. The potential for functional decline and poor outcomes is worsened by the backlog of elective procedures from the Covid-19 pandemic, which has extended NHS waiting times for joint replacements [67–71]. These waiting times may persist until 2031, as capacity has not recovered since the pandemic [72]. People that wait longer for their operation have higher healthcare resource use [73], with one UK study finding increased opioid use in post-pandemic joint replacement patients [74]. Hence, optimising health through pre-operative education to encourage physical activity could reduce healthcare costs, help manage symptoms, and improve longer-term outcomes. More interventions that focus on overcoming barriers to physical activity need to be robustly developed and tested to understand which approaches provide the most effective support for people awaiting joint replacement in the short and long-term.

### Limitations

Overall, findings from this review are confined by the small number of studies included in both the content and meta-analysis. Sample sizes within the retrieved studies were also considerably small, thereby limiting the statistical power of the quantitative analysis and limiting the generalisability of findings. Selection bias many have been introduced due to the exclusion of non-English studies from searches; this decision was taken as the qualitative analyst was only fluent in English. They were therefore only able to categorise content described in English. To serve the study purpose of capturing a range of PSE intervention content, we did not limit studies by intervention duration. To conduct the content analysis, intervention descriptions were broken down into content areas for PSE and exercise, based on available published data. Any further content and messaging delivered by therapists is unknown, due to lack of fidelity evaluations. Further, there was a lack of reporting clarity in some intervention descriptions, particularly for physiotherapy components, making intervention coding for the content and meta-analysis challenging. Only three programmes presented qualitative evaluation from participants, hence the synthesis of barriers and facilitators to intervention engagement is limited. A classical approach to cNMA was utilised to evaluate effectiveness, lumping PSE components together to provide overall summary measures across RCTs, and non-randomised studies. It was not appropriate to conduct a GRADE assessment to evaluate certainty of evidence, due to the small number of RCTs included in the meta-analysis, and therefore no conclusions can be drawn about the certainty of the evidence on effectiveness. The meta-analysis included small studies with high heterogeneity; study effects were significant but small. Therefore, results relating to the effectiveness of intervention components should be interpreted cautiously.

## Conclusions

It is important that people with osteoarthritis, particularly those awaiting joint replacement, remain physically active to prevent exacerbated pain and physical de-conditioning, which can negatively impact post-operative recovery. The current review highlights the potential effectiveness of various components of PSE for promoting exercise and improving psychological outcomes for people with knee osteoarthritis. Further research is needed to evaluate whether these improvements translate to longer-term positive impacts on physical functioning. PSE content should be delivered using relatable examples to enhance comprehension, alongside flexible, tailored exercises to ensure accessibility. This review highlighted the need for further research to co-design tailored and accessible PSE and exercise interventions and evaluate their effectiveness in RCTs with longer-term follow-up.

## Supplementary Information

Supplementary Material 1.

Supplementary Material 2.

Supplementary Material 3.

Supplementary Material 4.

Supplementary Material 5.

## Data Availability

All data generated or analysed during this study are included in this published article and its supplementary information files.

## Abbreviations

TKR: Total Knee Replacement
THR: Total Hip Replacement
NHS: National Health Service
PSE: Pain Science Education
RCT: Randomised controlled trial
cNMA: Component network meta-analysis
