# Supplementary material for: Pain science education and exercise interventions for people with knee or hip osteoarthritis: a systematic review, content and meta-analysis
Source: BMC Musculoskelet Disord. 2025 Nov 22;26:1092. doi: 10.1186/s12891-025-09313-4 (PMC12699862; doi:10.1186/s12891-025-09313-4)
Supplement: Supplementary file 1 — Supplementary Material 1. [file 12891_2025_9313_MOESM1_ESM.docx]

## Additional File 1. Search Strategy

Example database search strategy for articles investigating Pain Science Education (PSE) and exercise programmes for people waiting for hip or knee replacement, using Medline via Ovid.

| # | Note | Searches |
| --- | --- | --- |
| 1 | Population | exp osteoarthritis/ |
| 2 |  | osteoarthr$.tw. |
| 3 |  | ((degenerative or end-stage or painful or severe) adj2 arthritis).tw. |
| 4 |  | arthrosis.tw. |
| 5 |  | arthroplasty, replacement, hip/ |
| 6 |  | arthroplasty, replacement, knee/ |
| 7 |  | ((hip or knee) adj5 (replac* or operat* or surg* or arthroplast*)).tw. |
| 8 |  | or/1-7 |
| 9 | Intervention | ("pain science" or "therapeutic neuroscience" or "pain neuroscience" or "explain pain" or PSE or PNE).tw. |
| 10 |  | 8 and 9 |
| 11 |  | limit 10 to english language |
