# Supplementary material for: Pain science education and exercise interventions for people with knee or hip osteoarthritis: a systematic review, content and meta-analysis
Source: BMC Musculoskelet Disord. 2025 Nov 22;26:1092. doi: 10.1186/s12891-025-09313-4 (PMC12699862; doi:10.1186/s12891-025-09313-4)
Supplement: Supplementary file 4 — Supplementary Material 4. [file 12891_2025_9313_MOESM4_ESM.pdf]

Additional File 2.

Supplementary Table. Content summary of exercise component categories (N = 16) included with identified PSE and exercise interventions (N = 10).

Author (year)	Pilates sessions (group)	Blended group exercise sessions (aerobic/ resistance/ balance/ neuromuscular/ stretch)	Neuromuscular exercise sessions (group)	Breathing exercises	Functional home exercise: progressive walking	Functional home exercise: Chair squat/sit-to-stand/wall squat	Functional home exercise: Step up/down/ stepping	Functional muscle retraining (tailored using EMG profile)	Functional home exercise: knee flexion/ extension	Functional home exercise: quadriceps isolation	Functional home exercise: lunges	Functional home exercise: pelvic lifts	Functional home exercises: abdominal (e.g. plank)	Functional home exercise: Heel raises	Hamstring stretches	Calf stretches
Stanton et al. (2020)																
Stanton et al. (2021)																
Modarressi et al. (2023)																
Rabiei et al (2023)																
Preece et al. (2021)																
Supe et al. (2023)																
Terradas-Monllor et al. (2023)																
Louw et al. (2019)																
Lluch et al. (2018)																
Gholami et al. (2023)																
Count	1	1	1	4	3	5	4	1	4	2	2	2	2	1	1	1
