# Supplementary material for: Pain science education and exercise interventions for people with knee or hip osteoarthritis: a systematic review, content and meta-analysis
Source: BMC Musculoskelet Disord. 2025 Nov 22;26:1092. doi: 10.1186/s12891-025-09313-4 (PMC12699862; doi:10.1186/s12891-025-09313-4)
Supplement: Supplementary file 5 — Supplementary Material 5. [file 12891_2025_9313_MOESM5_ESM.docx]

Additional File 5. Sensitivity analyses.

Two sensitivity analyses were conducted. In the first, we explored a different methodological approach by estimating effect sizes using post-intervention values only (Figures 1.1 to 1.5). Please note that it is not possible for before-and-after studies since both studies only had one group. In the most cases, the trends were similar, though treatment effects derived from post-intervention values showed more conclusive effects, especially in functional outcomes, pain catastrophising, and pain self-efficacy scales. However, this reflects the issue of not accounting for baseline values as we have seen some difference among baseline values between groups in the included studies, which is common for small sample sizes.

In the second sensitivity analysis, we followed the conventional synthesis approach by combining different types of PSE. We used a network meta-analysis approach within random-effect models (Figures 2.1 to 2.5). Most results were similar to the main analysis, showing that PSE had general additive effects on exercise, except for the pain catastrophising scale outcome, which showed high heterogeneity resulting in a wide confidence interval.


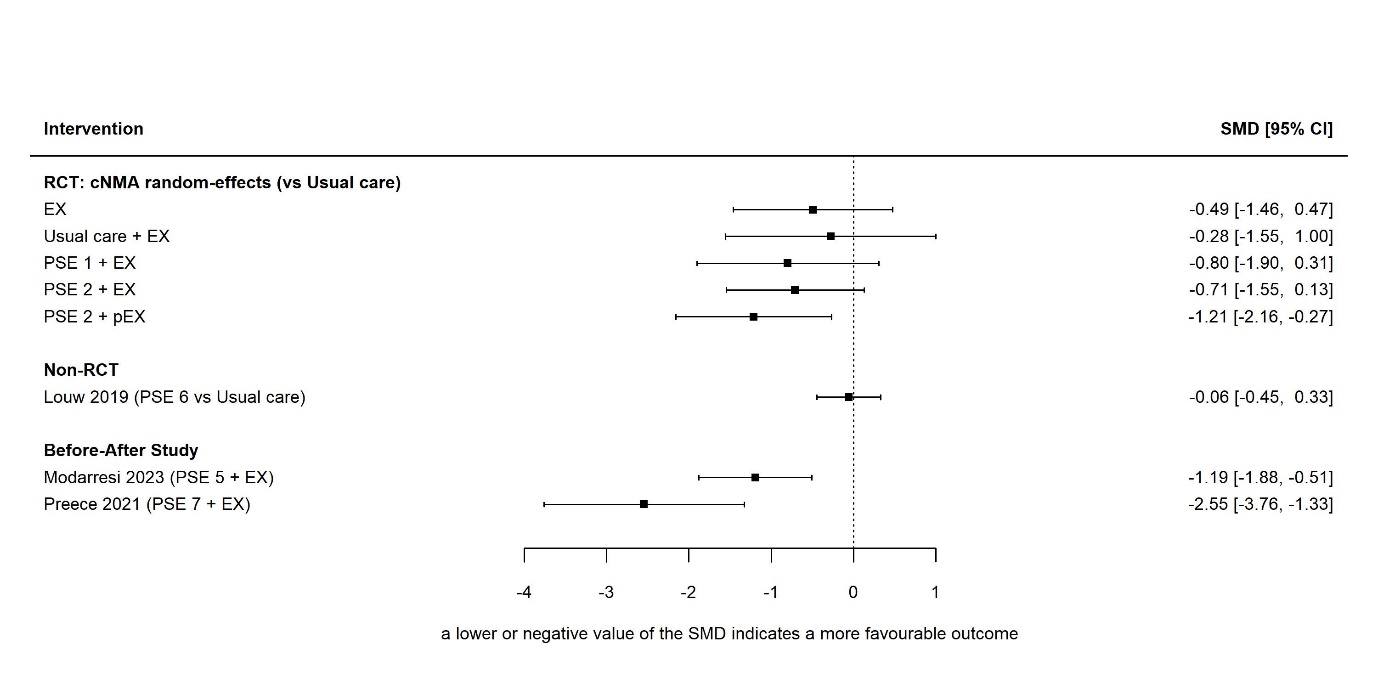


Figure 1.1: Sensitivity analysis: Forest plot (pain - WOMAC pain sub-scale)


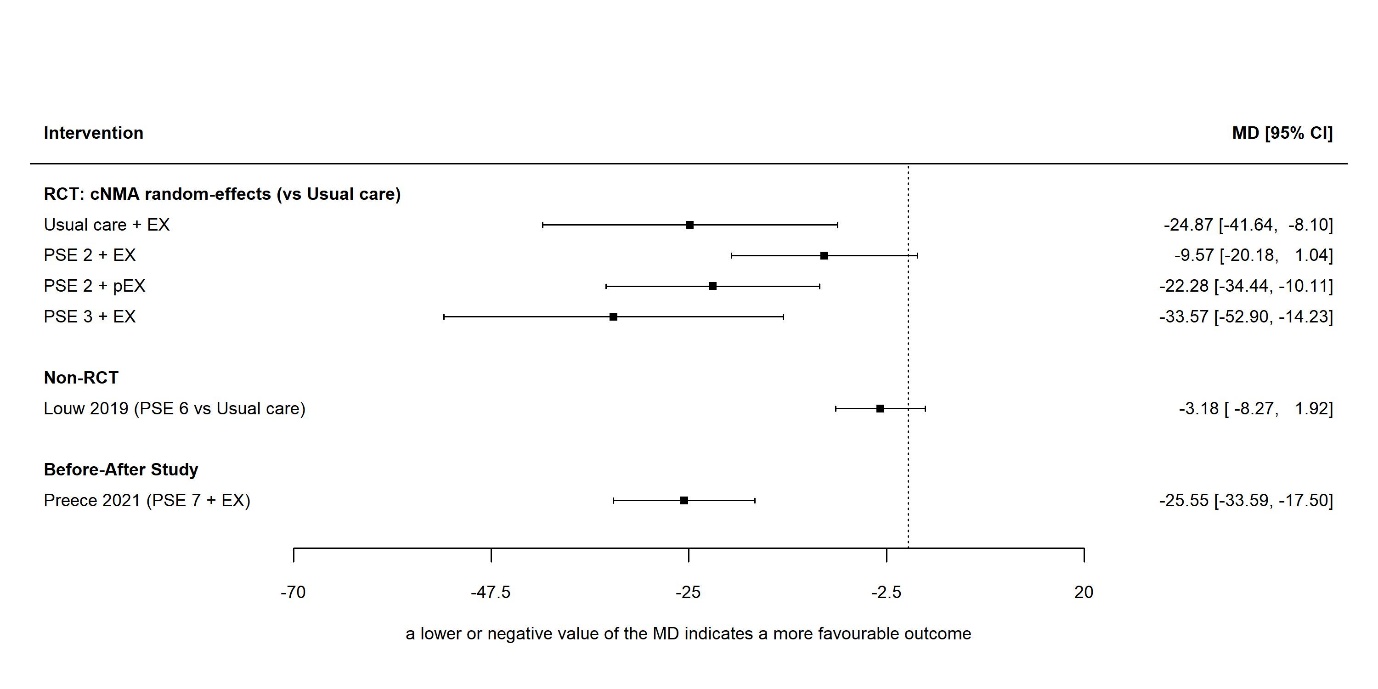


Figure 1.2: Sensitivity analysis: Forest Plot (physical functioning - WOMAC total score)


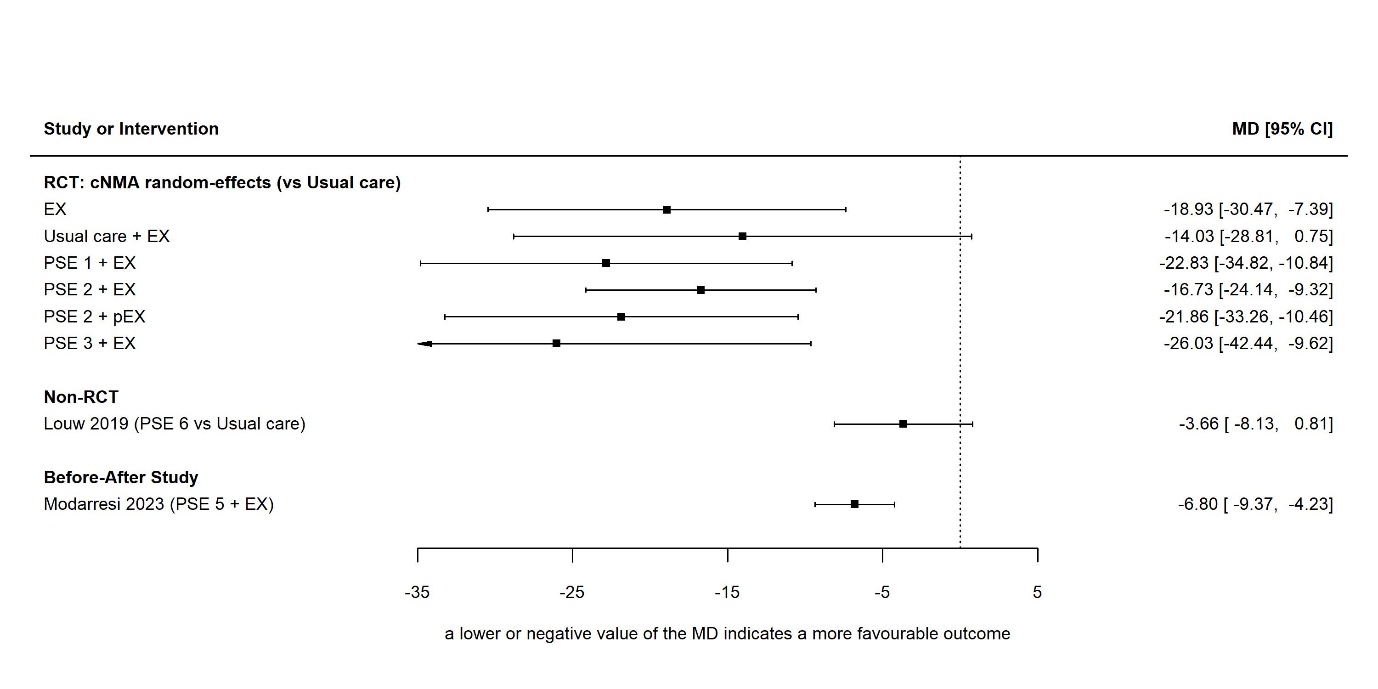


Figure 1.3: Sensitivity analysis: Forest plot (Pain Catastrophizing Scale)


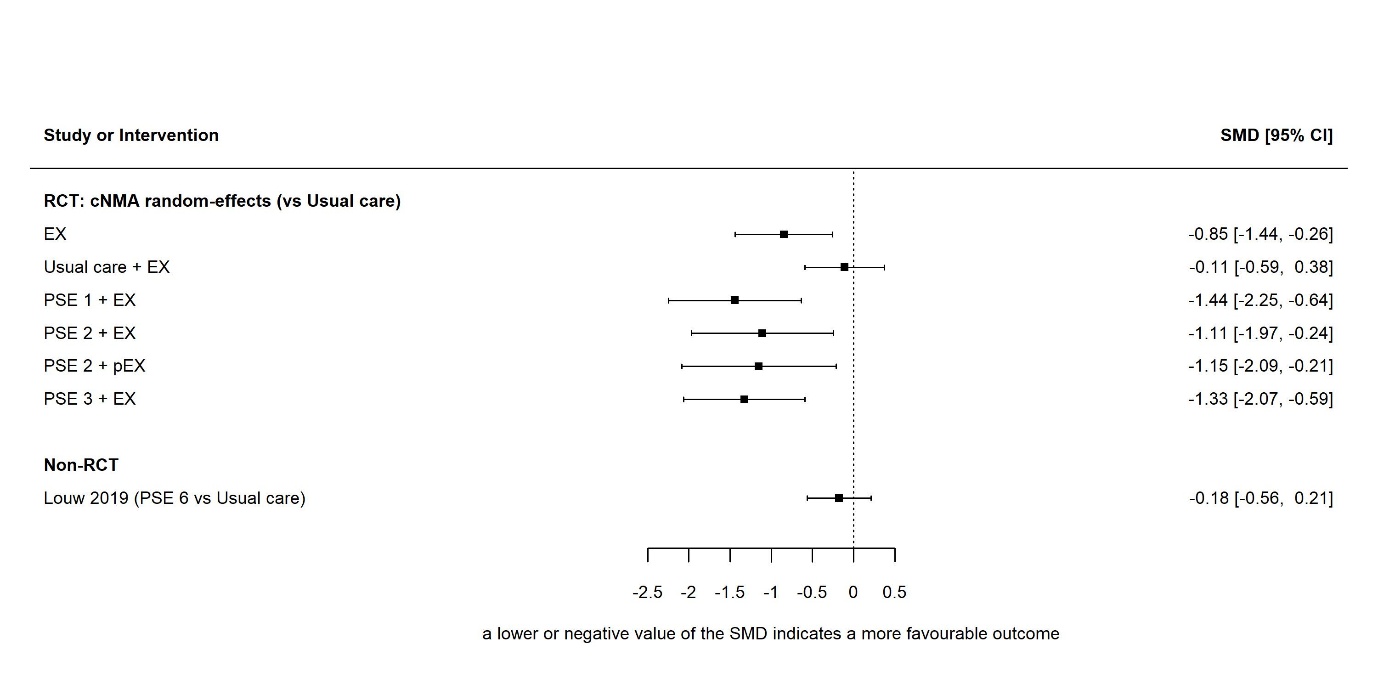


Figure 1.4: Sensitivity analysis: Forest plot (Tampa scale for kinesiophobia)


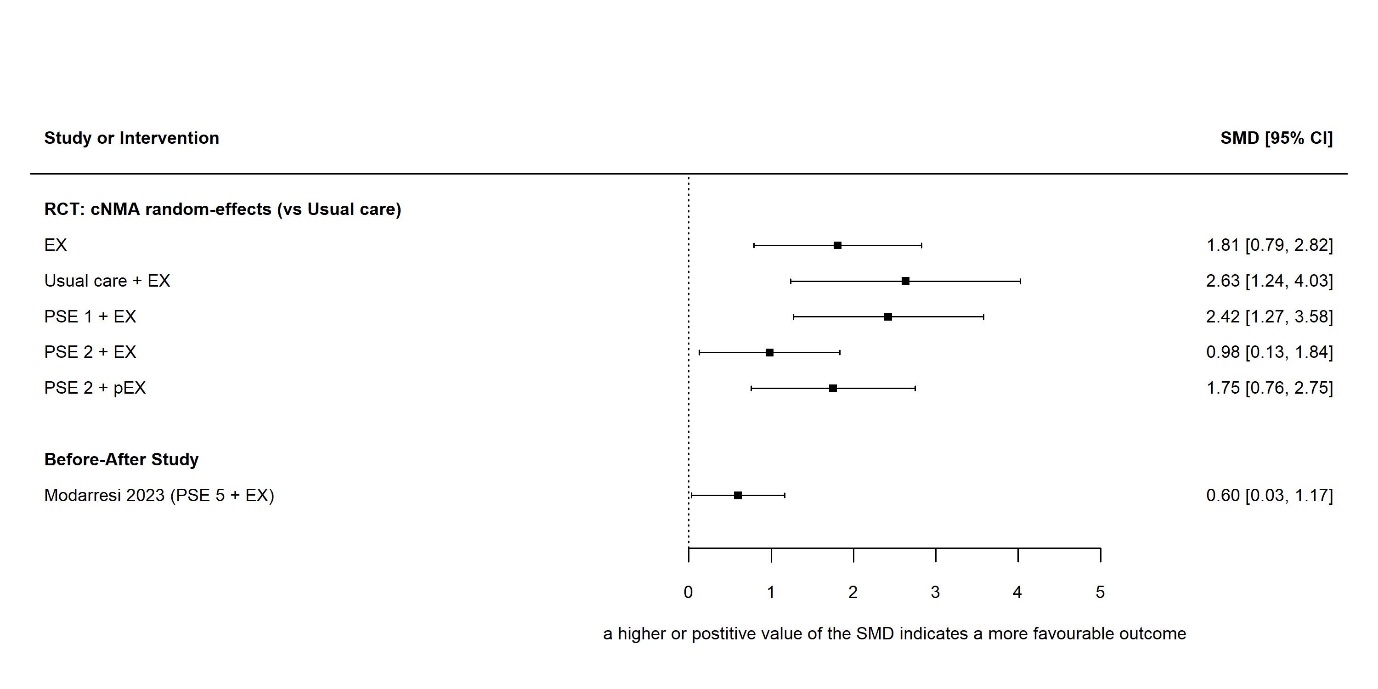


Figure 1.5: Sensitivity analysis: Forest plot (pain self-efficacy - Chronic Pain Self-Efficacy Scale or Pain Self-Efficacy Questionnaire)


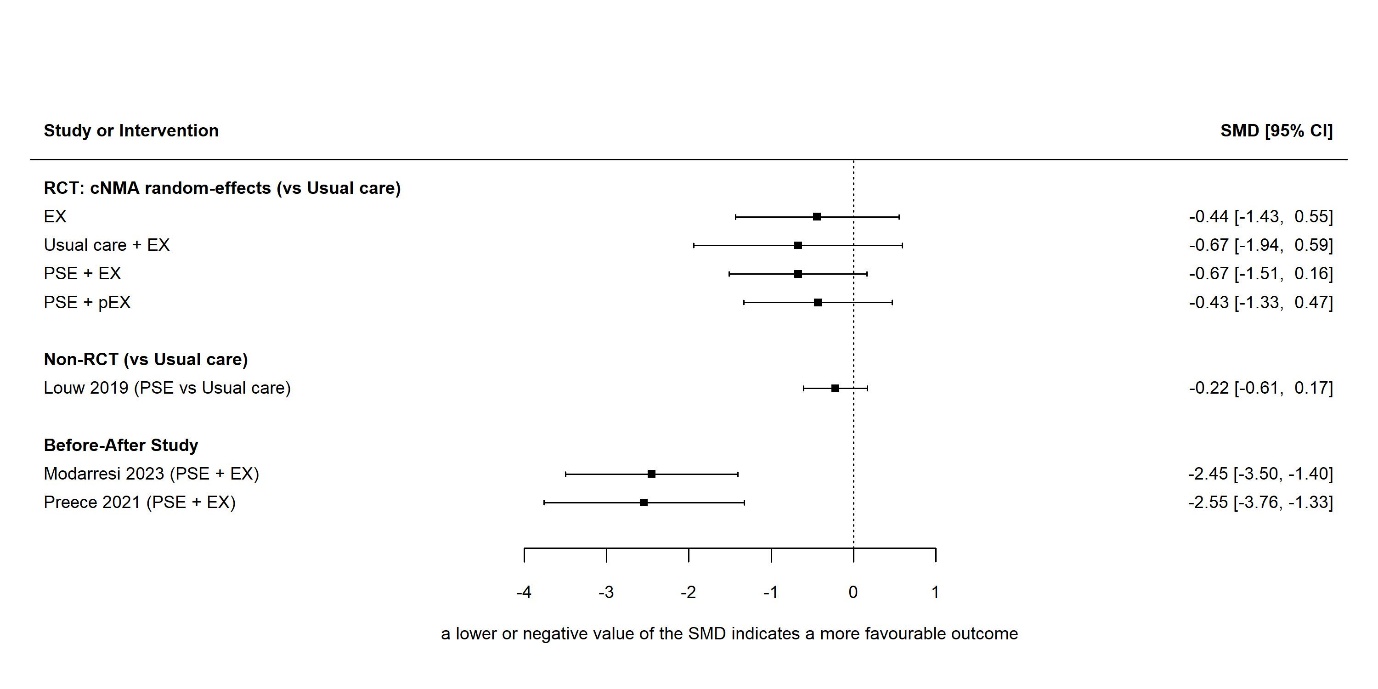


Figure 2.1: Forest plot of pain (WOMAC pain sub-scale): RCTs and observational studies by lumping different types of PSE


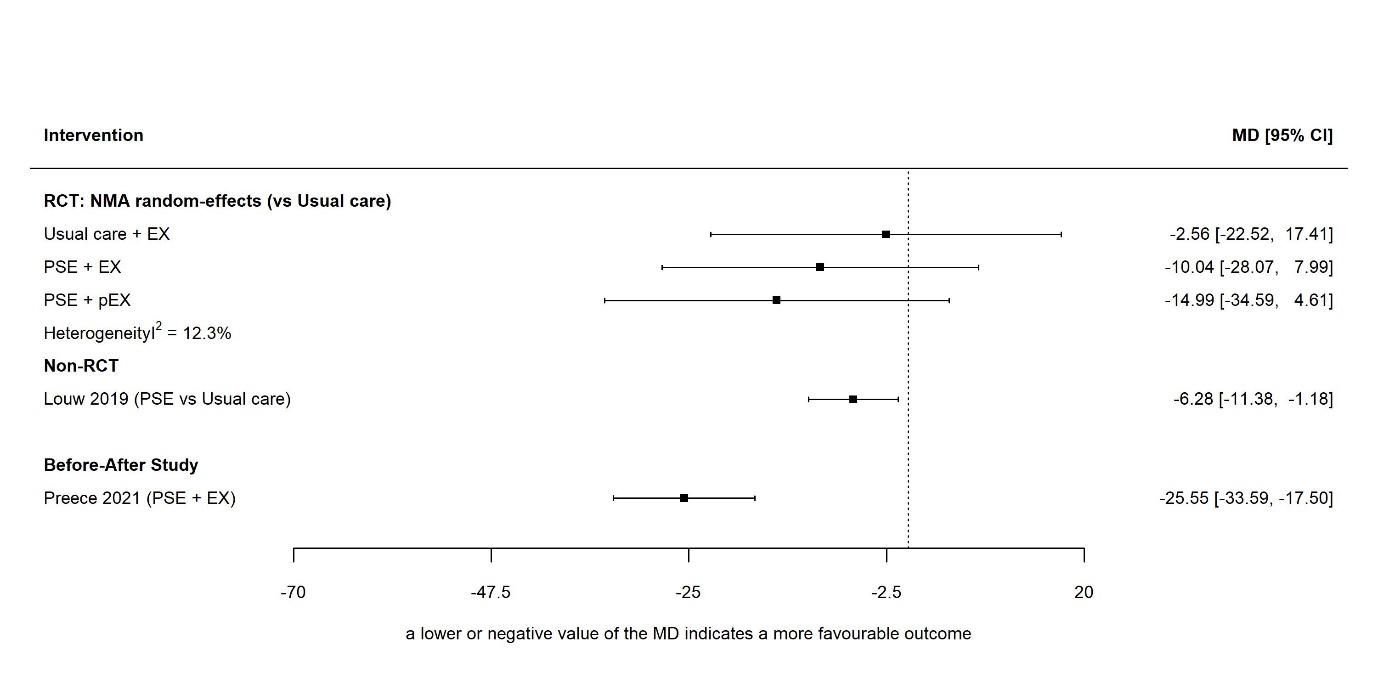


Figure 2.2: Forest Plot of physical functioning (WOMAC total score): RCTs and observational studies by lumping different types of PSE


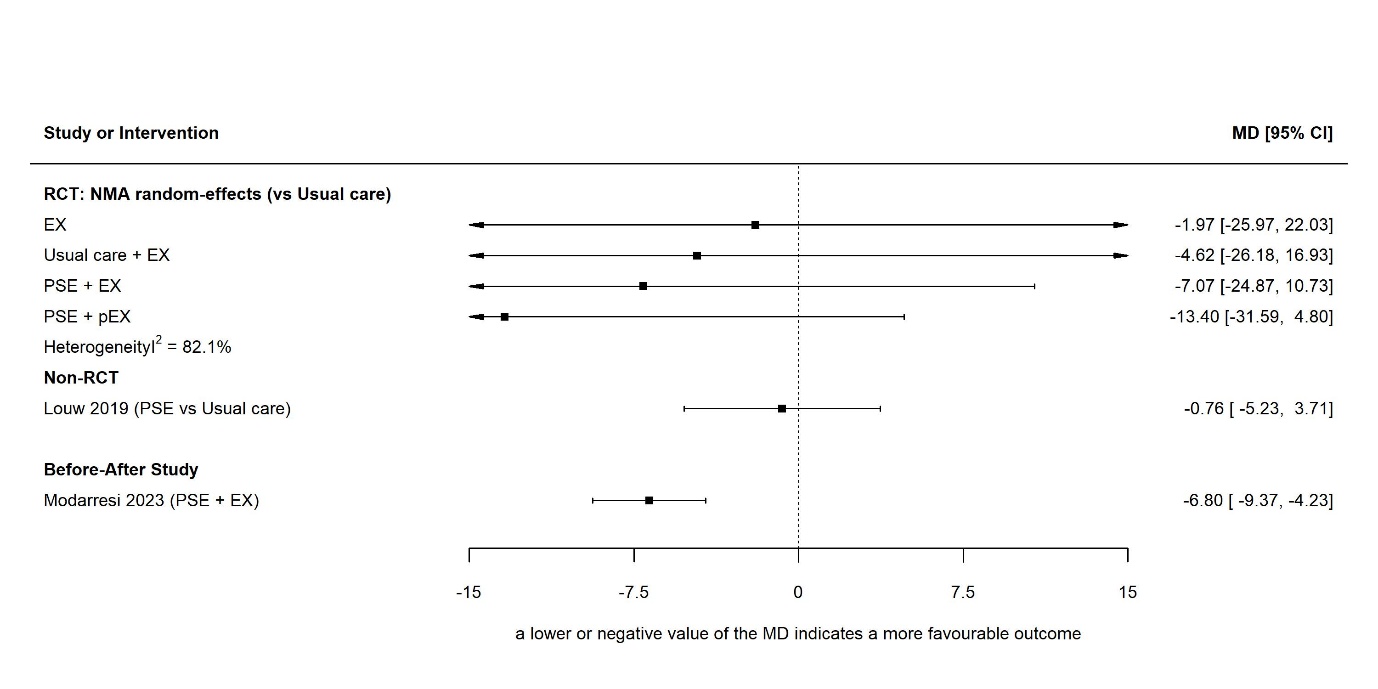


Figure 2.3: Forest plot of Pain Catastrophizing Scale: RCTs and observational studies by lumping different types of PSE


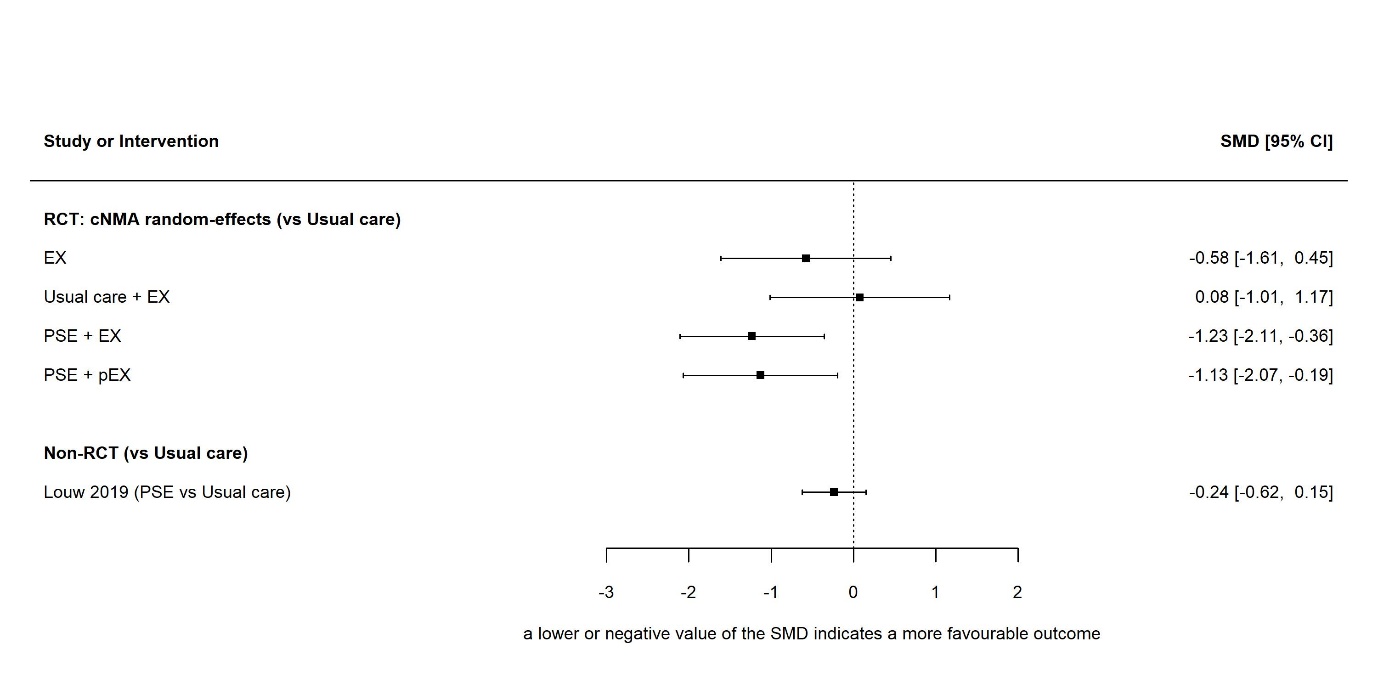


Figure 2.4: Forest plot of Tampa scale for kinesiophobia: RCTs and observational studies by lumping different types of PSE


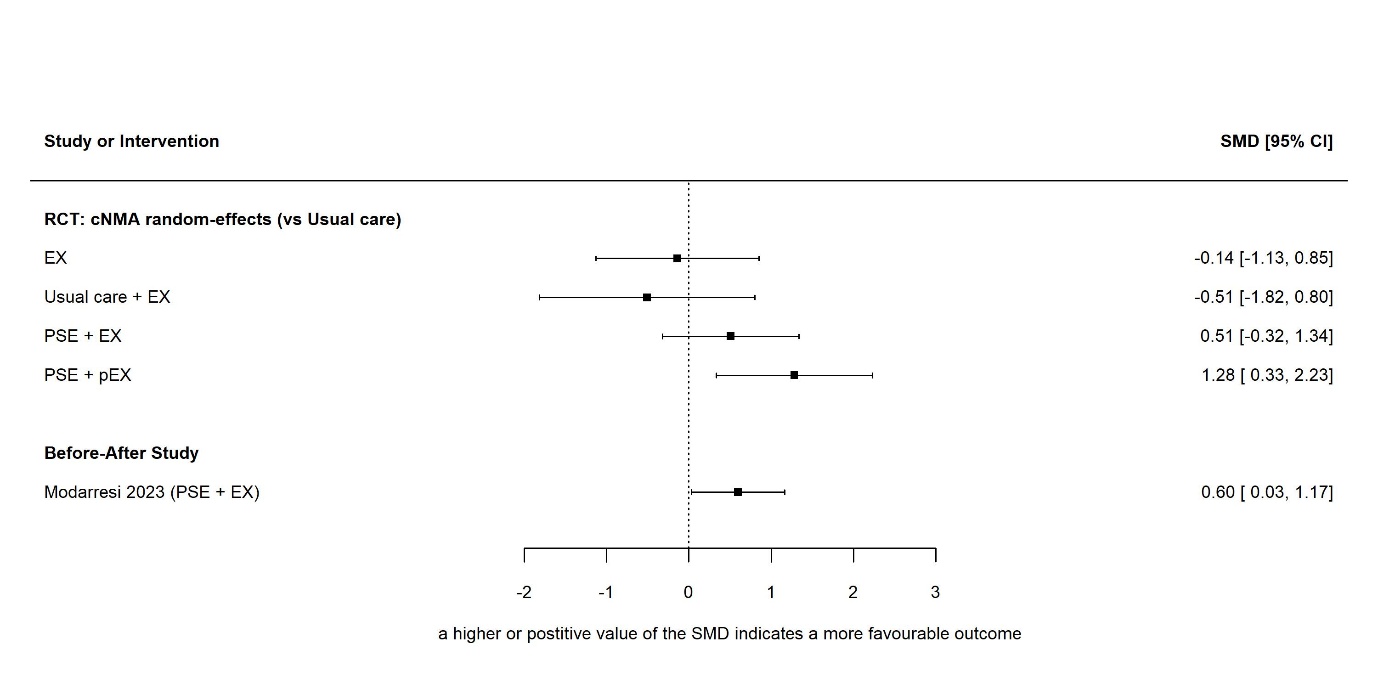


Figure 2.5: Forest plot of pain self-efficacy: RCTs and observational studies by lumping different types of PSE
